# Experience of Time and Subjective Age When Facing a Limited Lifetime: The Case of Older Adults with Advanced Cancer

**DOI:** 10.1177/08982643211063162

**Published:** 2021-12-30

**Authors:** Katsiaryna Laryionava, Anton Schönstein, Pia Heußner, Wolfgang Hiddemann, Eva C. Winkler, Hans-Werner Wahl

**Affiliations:** 1 9144 Heidelberg University Hospital, Heidelberg, Germany; 2 Martin Luther University Halle-Wittenberg (Saale), Germany; 3 Network Aging Research, 9144 Heidelberg University, Heidelberg, Germany; 4 Oncological Center Oberland, Hospital Garmisch-Partenkirchen, Germany; 5 9183 University Hospital, Ludwig-Maximilians University, Munich, Germany; 6 Network Aging Research and Institute of Psychology, 9144 Heidelberg University, Heidelberg, Germany

**Keywords:** geriatric oncology, time perception, subjective age, distress, quality of life, depression, advanced cancer

## Abstract

**Objectives:**

We addressed two questions: (1) Does advanced cancer in later life affect a person’s awareness of time and their subjective age? (2) Are awareness of time and subjective age associated with distress, perceived quality of life, and depression?

**Methods:**

We assessed patients suffering terminal cancer (OAC, *n* = 91) and older adults free of any life-threatening disease (OA, *n* = 89), all subjects being aged 50 years or older.

**Results:**

Older adults with advanced cancer perceived time more strongly as being a finite resource and felt significantly older than OA controls. Feeling younger was meaningfully related with better quality of life and less distress. In the OA group, feeling younger was also associated to reduced depression. Perceiving time as a finite resource was related to higher quality of life in the OA group.

**Discussion:**

Major indicators of an older person’s awareness of time and subjective aging differ between those being confronted with advanced cancer versus controls.

## Experience of Time and Subjective Age when Facing a Limited Lifetime: The Case of Older Adults with Advanced Cancer

Geriatric oncology has recently gained a lot of momentum as a discipline relevant both to clinical practice and to behavioral aging science (White et al., 2019). Two thirds of all new cancer cases are diagnosed in adults over the age of 60 years (Weir et al., 2015). Thus, we concentrate on two questions that have been addressed only on the margins of previous research: (1) Does life-threatening cancer in later life have any impact on one’s awareness of time and subjective age? (2) Do differences between individuals in their perception of time and discrepancies between their subjective and chronological age show any connection with key psychological outcomes such as distress, perceived quality of life, and depression?

### Research Background

A general finding is that cancer significantly impacts patients’ perceptions of time horizons independent of their chronological age (Fitzpatrick et al., 1980; Lövgren et al., 2010; Rasmussen & Elverdam, 2007; Rovers et al., 2019; van Laarhoven et al., 2011; Zhou et al., 2018). Previous research has shown, for example, that awareness of one’s limited time increases with a diagnosis of malignant disease and that cancer patients tend to reflect more on their lifetime, perceiving that time as a finite resource (Rasmussen & Elverdam, 2007). Simultaneously, *old age* has been characterized as being to a large extent driven by an increasing awareness that one’s future time perspective is becoming more and more limited (Carstensen, 2006). At the same time, older adults in general tend to feel younger than they chronologically are (Pinquart & Wahl, 2021). However, to the best of our knowledge, no study has yet compared the personal perceptions of time and subjective age in older adult patients suffering from (advanced) cancer against the perceptions of older adults not suffering from any life-threatening disease.

**Perception of time in later life**: **The role of cancer**: One’s perception of time and of increasing limitations on the perceived time remaining, hence future time perspective, is a core issue in socioemotional selectivity theory (e.g., English & Carstensen, 2016). Socioemotional selectivity theory states that as a person’s age increases, they perceive time as a finite resource, a change of perspective that influences priorities in selecting, for example, social goals. Goals with emotional meaning, as for instance, investments in intimate social relations become more important, while goals such as knowledge acquisition and living novel experiences tend to lose meaning (Carstensen, 2006). Importantly, according to socioemotional selectivity theory, lifespan-related shifts in personal priorities are due to a narrowed future time perspective rather than to chronological age. Supporting evidence for this comes from studies involving young adults: If future time perspective is constrained either experimentally or due to specific events (to the prospect of a move or to HIV infection, for example), younger people tend to express similar preferences to their older peers in favor of maintaining and enhancing intimate social relations (Fredrickson & Carstensen, 1990; Fung et al., 1999).

On the perception of remaining lifetime as an issue in cancer patients, one exploratory qualitative study involving 23 cancer survivors has shown that they experience some disruption of time continuity, showing increasing awareness, reflection, and prioritization of time (Rasmussen & Elverdam, 2007). Altered perceptions of time have also been used as an indicator of psychological distress and depression in palliative care among patients at the end of their lives (Julião et al., 2013). Qualitative research involving 12 patients with terminal cancer demonstrated that perception of time changes near the end of life, with the past becoming the dominant period of time in one’s mind (Rovers et al., 2019). Another study which included 96 disease-free and 63 patients with advanced cancer has shown that time perception may be an important factor in the distress suffered by cancer patients: a tendency to focus on the past and a slowdown in time perception were both correlated with distress (van Laarhoven et al., 2011). It has also been found that the perception of one’s future as being limited can lead to mental disorders and to increased fear of cancer recurrence (Zhou et al., 2018).

However, next to nothing is known about the perception of time among *older* adults suffering a life-threatening disease like advanced cancer. Socioemotional selectivity theory suggests that older adults suffering from cancer will likely experience their personal future as being more limited than older adults without life-threatening disease. Still, we are aware of only one relatively old study that provides support for this prediction (Fitzpatrick et al., 1980). In addition to perceiving future time as a limited resource, this paper also considers other key time-related parameters based on already existing conceptual and empirical works that might be particularly relevant to older adults with cancer. Zimbardo and Boyd (2015) argued that time orientation represents an important individual-difference variable. For example, the tendency to be more oriented toward the future than toward the past as a basic personal disposition may help cancer patients to generate the resources (including social contacts and leisure activities, for example) they may need to help them cope with their disease. Further, a classic and at least to some extent empirically confirmed prediction in the time research literature is that the passing of time tends to be experienced as running more quickly in old age than in earlier life (John & Lang, 2015). To our knowledge, no study so far has tested either whether this also applies to older cancer patients too, or whether, on the contrary, their disease burden could even be associated with an experience of time as passing more slowly (after all, one might well expect that long sequences of medical treatment and decreased ability to enjoy leisure activities will cause time to drag). Next, socioemotional selectivity theory (Carstensen, 2006) also suggests that the tendency to become increasingly aware of one’s lifetime is a fundamental aspect of the aging process. It seems natural to believe that this process may become even more pronounced in cases of cancer in later life. Finally, how people use their daily time is a long-established issue in gerontology or to put it differently, the need to use one’s now “free” time meaningfully after retirement is seen as one of the major challenges of becoming older (Cutler & Hendricks, 1990; Klumb & Baltes, 1999). In this regard, previous research has shown that serious chronic disease impacts one’s strivings in how one uses time and in physical activity (Jowsey et al., 2012).

To conclude, we argue that adopting a more comprehensive and multidimensional consideration of time awareness in older adults with life-threatening disease may help to improve our understanding of individual patients’ subjective approach to the disease and pave the way towards promising new behavioral interventions. Such interventions might include helping patients to learn to focus on positive experiences in their past and to refocus their view of their present time of life in ways that promote active emotional regulation and enjoyable experiences (Laureiro-Martinez et al., 2017).

**Subjective experience of aging**: **The role of cancer**: A now classic indicator of how individuals experience their aging is *subjective age*, obtained by asking how old or young they feel irrespective of their chronological age (Diehl et al., 2014). It has been found that older adults on average feel about 20% younger than their chronological age; only a small minority report feeling older than their real age (Rubin & Berntsen, 2006). Aside from this, a substantial body of longitudinal research underscores the fact that the younger a person feels, the better their health will tend to be, at least over longer periods and after controlling for confounding influences. For instance, having a younger subjective age has been found to predict better functioning of one’s objective and subjective memory, better sleep quality, less overnight hospitalization, fewer occurrences of dementia, and lower mortality due to all causes (Hülür et al., 2015; Stephan et al., 2016, 2018; Sun et al., 2017). The meta-analyses available further substantiate this role of subjective age in predicting good health across a range of health outcomes (Alonso Debreczeni & Bailey, 2020; Westerhof et al., 2014).

It may be that experiencing life-threatening cancer in old age narrows the gap between subjective and objective age or even increases the feeling of being older than one’s chronological age as compared to peers in good health for the following reasons. First, the perception feeling younger has been interpreted as an act of distancing from one’s aging that may help in maintaining well-being and identity (Wurm et al., 2017). The task of acting on such an adaptational strategy is likely to become more difficult and to require more effort in situations when the older individual is confronted with a life-threatening illness like cancer. Second, research provides support for the idea that older adults with better physical fitness and suffering less depression feel younger than their less fit, more depressed peers (Bergland et al., 2014). Cancer patients report both reduced physical fitness as well as increased depression (Smith, 2015; Wilson et al., 2007). Third, in one particular study, a heavier burden of daily stress, as assessed using an intensive data-collection format over several days, has been found to be linked with older subjective age (Bellingtier et al., 2015). Cancer patients tend to report more daily distress due to disease burden as well as more pain, factors that may have an impact on their subjective feeling of age (Hurria et al., 2009; Sabatini et al., 2021).

In consequence and taking the health-relevant findings of past research into account, feeling younger than one’s chronological age may represent an important resilience factor even when confronted with the burden of a disease like cancer. Some research in the area provides empirical support for this assumption. Boehmer (2006) found that older cancer patients who felt younger than their chronological age showed better psychosocial adaptation in that such patients report lower levels of perceived disability and avoidance-oriented coping and higher levels of satisfaction with recovery, self-efficacy, and meaning-focused coping than individuals who report a higher subjective age. In addition, Martin et al. (2019) found that older adults with cancer revealed a more negative attitude toward their aging and that more negative attitudes toward aging also go together with lowered physical and mental functioning in older adults both with and without cancer. Still, more research remains to be done to provide a better understanding of the role of subjective age in older adults faced with severe cancer.

### Research Questions and Hypotheses

The first objective of this study is to compare the perception of time among older patients with advanced cancer against that of older adults free of life-threatening disease. We hypothesize that older adults with life-threatening cancer will tend to experience their future time as a limited resource more strongly than will the controls (H1). We also examine at the exploratory level, if any differences can be found between the two groups in relation to the four other time dimensions that we consider. Second, we hypothesize that subjective age of older cancer patients, as well as the percentage of those who feel the same age or older than their chronological age, will be higher than the same figures for the controls (H2). Third, we predict that higher scores in experience of one’s lifetime as a finite resource and higher subjective age will be associated with heightened distress, lowered quality of life, and increased depression among older adults whether they suffer from cancer or not (H3).

## Method

### Study Design

We conducted a cross-sectional study among adults older than 50 years, one group diagnosed with advanced cancer (Older Adults with advanced Cancer - OAC) and one group free of it, thus serving as an older adult control sample without life-threatening disease (OA). All cancer patients had advanced metastatic hematological/oncological neoplasia (at disease stage III/IV) and with decisions to limit treatment being either under discussion or already determined.

Older adults with advanced cancer were recruited at the Department of Hematology and Oncology at the University Hospital in Munich and included in the study based on the following criteria: a decision to terminate anti-cancer treatment, sufficient knowledge of German language, absence of any serious cognitive impairment based on the clinical opinion of a physician, and written informed consent. Ethical approval was obtained from the Ethics Commission of the University Hospital in Munich.

OA were recruited based on the infrastructure of the Department of Psychological Aging Research of University of Heidelberg. Inclusion criteria were as follows: chronological age ≥50 years old, no severe disease, sufficient knowledge of German language, absence of any cognitive impairment, and written informed consent. Ethical approval for the study was obtained by the ethical board of the Faculty of Behavioral and Cultural Studies of Heidelberg University.

### Sample Description

**Older adults with advanced cancer**: A total of 336 OAC patients were screened for eligibility. Out of 129 eligible patients, 97 agreed to participate in the study. The main reasons given by the 32 patients for not participating was that they were not feeling physically and mentally able to answer a long list of structured questions. Questionnaires for which <60% of the items had been completed were excluded from the analysis (*n* = 5). Finally, a total of 92 patients were selected for analysis.

Thirty-five patients (38%) had hematological diseases and 57 (62%) had solid tumors. For a large majority of the patients (*n* = 78 or 85%), decisions had been made to limit treatment (i.e., they were not to be given cardio-pulmonary reanimation and/or were not to be transferred to an intensive care unit). The average age of the OAC sample was 70.95 (*SD* = 8.5; with a range of 51–92 years. 53 (58%) of the participants were female and 68 (74%) were married.

**Older adults without life-threatening disease**: To recruit the OA comparison group, we randomly drew *N* = 100 individuals aged 50 years and older from an existing sample of *N* = 423 individuals aged 40 years and older, who had been originally assessed as part of the project “Awareness of Age-Related Change: A Cross-Cultural Cooperation.” The original sample included only adults who described themselves as “quite healthy,” a fact reflected in their average subjective rating of 5.24 (*SD* = .85) based on a scale from 1 (“poor”) to 6 (“excellent”). The sample size and a lower age threshold of 50 years was selected to make the group approximately similar to the OAC group. Due to 11 individuals declining to participate, the final sample of the OA group totaled *N* = 89 subjects. The average age of the OA sample was 67.98 (*SD* = 11.5; the age range was 50–92 years), and 46 (52%) participants were female. 62 participants (70%) were married, while information on the marital status was missing for four participants (5%).

A comparison of the OAC and OA groups in terms of available demographic information can be found in Supplementary Table 1.

### Measurements

Due to the great vulnerability of our OAC clinical sample, we reduced the data assessment burden to a minimum, which resulted in using predominantly established 1-item measures.

**Experience of Time**: First, to address a core issue of socioemotional selectivity theory (Carstensen, 2006), hence that people experience their future time perspective increasingly as a limited resource as they age, we used an item with the following phrasing: “I often think that time is a finite resource in my life.”^1^ Second, following Zimbardo and Boyd’s (2015) argument that time orientation is an important variable of difference between individuals, we created an item that targets one major aspect of time orientation; whether a person is more future- or past-oriented, as follows: “I think more often about what will come rather than about time that I have lived till this day.” In a third item, we addressed the passing of time (John & Lang, 2015): “Time passes faster today than ever before in my life.” Fourth, again informed by socioemotional selectivity theory (Carstensen, 2006), one item was phrased as follows: “I am concerned with the topic of time in my life.” In a fifth item, we draw from the established literature on time use and aging along with literature on time use and disease (Cutler & Hendricks, 1990; Jowsey et al., 2012): “I think a lot about whether I use my time well.” The answering format used for each item was a Likert scale ranging from 1 (“completely disagree”) to 5 (“completely agree”).

**Subjective Age**: For subjective age, participants were asked to indicate the age they felt themselves to be at the moment of assessment in years. The exact phrasing was based on the established 1-item approach mostly used in similar research (Montepare, 2009; Rubin & Berntsen, 2006): “Some people feel older or younger than they actually are. Some people feel as old as they are. Fill in the age (in years) that you feel at this moment.” We calculated a score for proportional subjective age difference ([Subjective Age − Chronological Age]/[Chronological Age]) to represent the extent to which a person’s subjective age differed from their chronological age (e.g., a score of −0.20 would indicate that the person felt 20% younger than his or her chronological age) (Dutt et al., 2018; Rubin & Berntsen, 2006). Due to the unrestricted format, this proportional score can produce some unrealistic values which may heavily influence statistical models. We addressed this by setting observations further than 3 *SD*s from the mean (*n* = 3) to “missing” in our modeling approaches. These missing values were then handled by multiple imputation. For selected analyses, we also dichotomized subjective age based on the proportional score, classifying participants into either feeling younger or the same age/older.

**Assessment of Clinical Outcome Variables**: Level of distress was assessed using a German-language version of the National Comprehensive Cancer Network (NCCN) Distress Thermometer (Mehnert et al., 2006). The perceived quality of life was assessed using the question “How would you rate your overall quality of life during the past week?” and was measured using a 7-point Likert scale from 1 (“very bad”) to 7 (“excellent”) taken from the European Organisation for Research and Treatment of Cancer Quality of Life Questionnaire EORTC-QLQ-C30. Depression was assessed via the short Whooley Depression Scale (Whooley et al., 1997). The questionnaire included two questions on depressive mood and anhedonia: (1) “During the past month, have you often been bothered by feeling down, depressed, or hopeless?” and (2) “During the past month, have you often been bothered by a lack of interest or pleasure in doing things?” The answering format is Yes/No for both questions. Participants who gave an affirmative response to both questions were interpreted as “having depression.”

**Overall Assessment Procedure**: All assessments for OACs were completed using a face-to-face interview format by a physician trained in conducting studies. For OA, all assessments were done using a self-assessment format based on questionnaires sent to participants and returned by them using the pre-paid, pre-addressed envelopes provided.

### Statistical Analysis

To describe the study’s OAC and OA samples, we calculated means and *SD*s where the response was assessed on a continuous or discrete scale. We also calculated Pearson’s r to check for bi-variate correlations between the study variables.

Hypotheses 1 and 2: We used linear regression to compare perceptions of time and subjective age indicators (outcomes) between the OAC and OA participants (dichotomous predictor), controlling for participants’ available demographic characteristics (chronological age, sex, and marital status) and psychological burden (depression). Further, we used logistic regression to examine the association between the proportion of participants feeling younger (dichotomized outcome) and OAC/OA groups (dichotomous predictor), likewise controlling for the available demographic variables (chronological age, sex, and marital status) and depression.

For Hypothesis 3, we used hierarchical linear regression (for the outcomes distress and quality of life) and a logistic regression approach (outcome: depression). We included both theoretically relevant predictor variables, that is, “Time as a Finite Resource” and “Subjective Age” (using a proportional difference score). Due to the total sample size of patients and the controls available for this analysis, we considered only a minimum of confounding factors, that is, age, gender, and marital status. Overall, we tested three models: Model 1 included, besides the demographic control variables, the predictor variables “Time as a Limited Resource” as well as Subjective Age. We examined the interaction with the group membership (OAC vs. OA) in separate models, as power to show such interactions is in general more limited. The interaction term “Time as a Limited Resource” x “Group Membership” was included in Model 2a to detect whether potential effects of the predictor differed between the groups. Similarly, Model 2b included the interaction term “Subjective Age” x “Group Membership.” In case of a significant interaction, we carried out a stratified analysis. We did not test for triple interaction effects.

Missing values were rare (no variable with more than 3% of missings, see Supplementary Table 2). In calculations relevant for Hypothesis 1–3, missing data was handled by multiple imputation using chained equations (50 imputation datasets) (van Buuren & Groothuis-Oudshoorn, 2011).

Results were considered statistically significant at *p* < .05 (using two-sided tests). No multiplicity adjustments were used, and all statistical analyses were conducted using R version 3.6.1.

## Results

### Descriptive Data

Table 1 provides the mean and *SD* of each study variable as well as the inter-correlations given separately for OAC and OA groups, except for the dichotomously coded depression variable. As far as time-related variables are concerned, participants (in both OAC and OA) tended on average to locate themselves on the middle to higher range of the answer scale, with an empirical variation of between 3.1 and 4.1. Hence, participants overall tended to take a neutral stance or to agree with the statements “I often think that time is a finite resource in my life,” “I think more often about what will come rather than about time that I have lived till this day,” “Time passes faster today than ever before in my life,” “I am concerned with the topic of time in my life,” and “I think a lot about whether I use my time well.” Standard deviations for the two groups were quite similar, detailed descriptive comparisons are depicted in Supplementary Figure 1. Time-related variables revealed positive inter-correlations in both groups, with the strongest correlation amounting to *r* = .55 in the OA group between “Time as a finite resource” and “Time as current concern,” but most correlations were below .40. Hence, and as expected, the selected time-related variables likely grasp different time-related contents. The proportional subjective age-discrepancy score indicated that OAC participants felt on average 2% older than their chronological age, whereas OA felt on average 8% younger.

**Table 1.** Mean, SD, and Correlations [95% CI] between Variables Used in the Study: Older Adults with Cancer (OAC) Below the Diagonal and Older Adults without Life-Threatening Disease (OA) Above the Diagonal.

Variable OA OAC		*M*_OA_ *M*_OAC_	*SD*_OA_ *SD*_OAC_	1	2	3	4	5	6	7	8
1. “Lifetime lim. res.”		3.57	1.0	—	−.09	.32**	.55**	.24*	.09	−.06	.24*
		4.11	1.0		[−.29, .13]	.12, .50]	[.39, .68]	[.03, .43]	[−.12, .29]	[−.27, .15]	[.03, .43]
2. “Future vs past”		3.08	1.0	.20	—	.25*	.15	.21*	.10	.15	−.02
		2.95	1.2	[−.01, .39]		[.04, .43]	[−.06, .35]	[.00, .40]	[−.11, .30]	[−.07, .35]	[−.23, .19]
3. “Passing of time”		3.82	1.1	.25*	.25*	—	.33**	.20	.11	.17	−.12
		3.62	1.4	[.05, .43]	[.05, .44]		[.12, .50]	[−.01, .39]	[−.10, .32]	[−.04, .37]	[−.33, .09]
4. “Time as current conc.”		3.11	1.0	.36**	.15	.30**	—	.40**	.12	.17	.03
		3.24	1.2	[.17, .53]	[−.05, .35]	[.10, .48]		[.21, .56]	[−.09, .32]	[−.05, .36]	[−.18, .24]
5. “Using time”		3.07	1.0	.13	.18	.32**	.45**	—	.02	.21*	−.12
		3.10	1.3	[−.08, .33]	[−.03, .37]	[.13, .49]	[.27, .60]		[−.19, .23]	[.00, .40]	[−.33, .09]
6. Subj. age (prop. score)		−0.08	0.1	.11	−.21	−.11	−.02	−.18	—	.27*	−.32**
		0.02	0.2	[−.10, .32]	[−.40, .00]	[−.32, .10]	[−.23, .19]	[−.38, .03]		[.06, .45]	[−.49, −.11]
7. Distress		4.89	2.4	−.06	−.01	−.10	−.02	.09	.12	—	−.41**
		5.96	2.4	[−.26, .15]	[−.22, .19]	[−.30, .11]	[−.22, .18]	[−.12, .29]	[−.09, .33]		[−.57, −.21]
8. Self-assessed quality of life		4.99	1.2	−.14	.04	−.09	−.01	−.03	−.20	−.37**	—
		3.15	1.5	[−.34, .06]	[−.17, .24]	[−.29, .12]	[−.22, .19]	[−.23, .18]	[−.39, .02]	[−.53, −.17]	

*Note. M* and *SD* are means and standard deviations for the Older Adults without Life-Threatening Disease (OA; *n* = 89) and Older Adults with Cancer (OAC; *n* = 92) group. Values in square brackets indicate the associated 95% confidence interval for each correlation. Missing values handled by pairwise deletion. Depression not included as it was assessed dichotomously.

*p < .05, **p < .01.

With respect to clinical outcome variables, the distress level for OACs was *M* = 5.96 (*SD* = 2.4) and self-reported quality of life amounted to *M* = 3.15 (*SD* = 1.4). The distress level for OAs was *M* = 4.89 (*SD* = 2.4), a figure as expected below the one for OACs, while their self-assessed quality of life, at *M* = 4.99 (*SD* = 1.2), was higher than in the OAC group. Finally, *n* = 49 (53%) of OACs were likely depressed, whereas this could be said for only *n* = 18 of the OA participants (21%). Hence, OACs consistently revealed a heavier psychosocial burden.

### Testing H1: Cancer and Perception of Time

**Figure 1.**
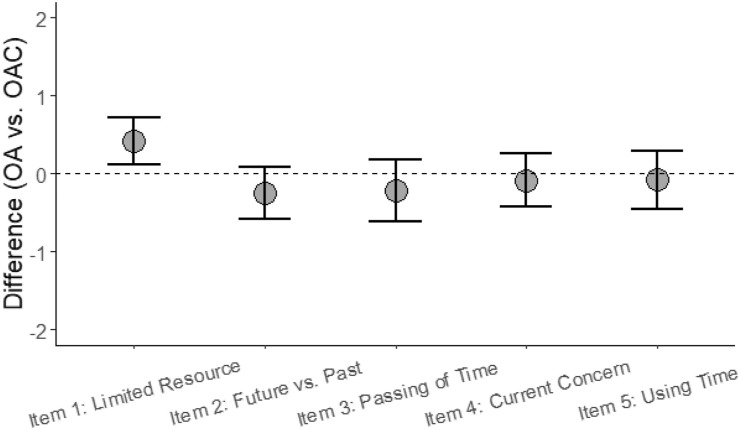
Comparing Experience of Time Indicators in Older Adults with and without Life-Threatening Disease. *Note.* Adjusted difference in agreement (y-axis, higher values indicate stronger agreement in OAC) between the older adults without terminal disease group (OA, *n* = 89) and the older adults with cancer group (OAC, *n* = 92) across time-related variables (x-axis). Dots represent mean differences, and error bars indicate the associated 95% confidence interval. Effects are controlled for participants’ age, sex, family status, and reported depression. The dashed reference line indicates a null effect. Missing data was handled by multiple imputation.

As displayed in Figure 1, when comparing the two groups across the five time variables, a significant difference in the agreement scores could only be shown for the “Life time as a limited resource”-Item: When controlled for age, sex, family status, and depression, OAC showed a somewhat higher agreement by about 0.41 score points (95% CI [0.11; 0.72]; *p* = .009). No significant differences between the two groups could be shown in the other four dimensions of perception of time that we considered.

### Testing H2: Cancer and Subjective Age

The majority of OACs either felt the same age (*n* = 33; 38%) or older than their chronological age (*n* = 28; 32%). Only 26 OACs (30%) indicated that they felt younger. In contrast, the majority of OAs reported feeling younger (70%, *n* = 62). 19 (21%) stated they felt the same age, and only eight older adults (9%) reported feeling older than their chronological age. Logistic regression revealed a significant inverse relationship between feeling younger (dichotomized: yes/no) and being in the OAC group, meaning that OAC were less likely to feel younger than OA, even when this effect was controlled for age, sex, family status, and depression (OR: 0.21; 95% CI [0.10; 0.42]; *p* < .001).

**Figure 2.**
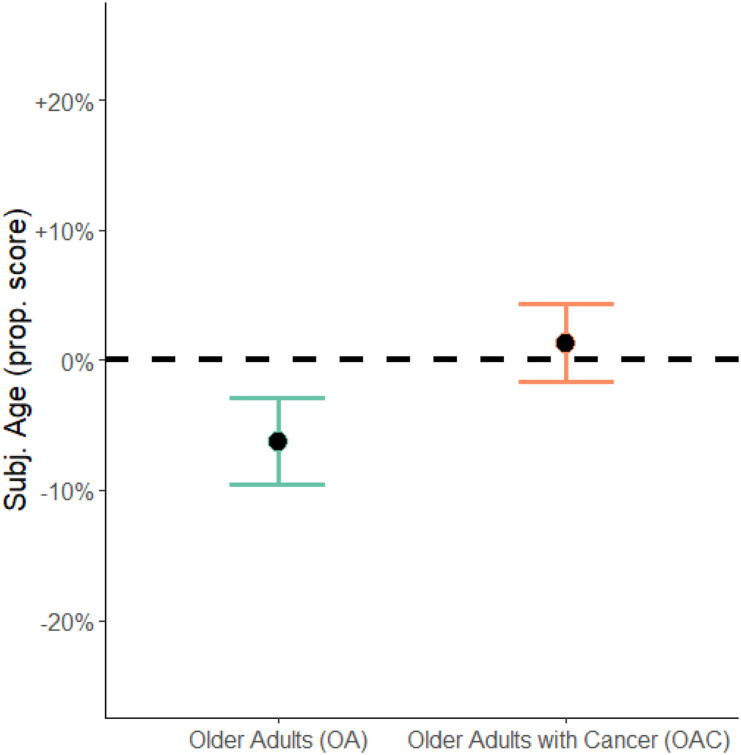
Comparison of Subjective Age (Proportional Score) in Older Adults with and without Life-Threatening Disease. *Note.* Participants’ mean subjective age (y-axis) as proportional score in older adults without life-threatening disease (OA, *n* = 89) and older adults with cancer (OAC, *n* = 92). Error bars represent 95% confidence intervals, estimates are controlled for age, sex, family status, and reported depression. The dashed reference line indicates subjective age equal to chronological age.

Further, the results revealed a significant difference in the (continuous) subjective age proportional score for OAC as compared to the OA group. Compared to OA, OACs subjective age proportional score was 8% points higher (95% CI: [0.04; 0.12], *p* < .001; see also Figure 2), meaning that OACs had a stronger tendency to feel older.

For illustration purposes, Supplementary Figure 2 shows the distributions in proportional scores for subjective age among OACs and OAs along the chronological age continuum. One can see that the overlap in terms of subjective age between the groups is quite limited and that a considerable proportion of those in the OAC group lie either on or above the zero-line, indicating that they feel the same age or older than their date of birth suggests. In contrast, a considerable portion of the OAs lie below the zero-line all along the entire chronological age continuum.

### Testing H3: Associations between Time as a Limited Resource and Subjective Age to Distress, Quality of life, and Depression

Results of hierarchical linear regression for the outcomes distress and quality of life are summarized in Table 2. Looking at participants’ self-rated distress as outcome, older subjective age was associated with higher distress. A dependency of this effect based on the cancer status (OA/OAC) as captured by the respective interaction term could not be shown. Subjective age appeared to play a similarly strong role in explaining participants’ distress as the cancer status. No association between the perception of time as a finite resource and participants’ distress could be shown.

**Table 2.** Predicting Distress and Quality of Life, Based on Demographic Variables, Time as a Finite Resource, Proportional Subjective Age Score, and Group Membership (OA vs. OAC).

	Distress			Quality of life		
Predictor	Model 1	Model 2a	Model 2b	Model 1	Model 2a	Model 2b
	ß [95% CI]	ß [95% CI]	ß [95% CI]	ß [95% CI]	ß [95% CI]	ß [95% CI]
Age	−.14 [−.29; .00]	−.14 [−.29; .00]	−.14 [−.29; .00]	−.05 [−.18; .07]	−.07 [−.19; .05]	−.06 [−.18; .07]
Sex	.16* [0.02; 0.30]	.16* [0.02; 0.31]	.15* [0.01; 0.30]	−.05 [−.17; .07]	−.08 [−.20; .04]	−.05 [−.17; .07]
Family status (alone vs. with partner)	.04 [−.11; .18]	.04 [−.11; .18]	.04 [−.11; .18]	.03 [−.09; .15]	.02 [−.09; .14]	.03 [−.09; .15]
Group (OA vs. OAC)	.21** [.05; .36]	.21* [.05; .36]	.20* [.04; .35]	−.50*** [−.63; −.37]	−.50*** [−.63; −.38]	−.49*** [−.62; −.36]
Time as a finite resource	−.06 [−.21; .09]	−.06 [−.21; .09]	−.06 [−.21; .09]	.04 [−.08; .17]	.06 [−.07; .18]	.04 [−.08; .17]
Subj. age (prop. score)	.19* [.04; .35]	.19* [.04; .35]	.21** [.06; .37]	−.22** [−.35; −.09]	−.22*** [−.35; −.09]	−.24*** [−.37; −.10]
Group x Time as finite resource	—	.01 [−.14; .15]	—	—	−.17** [−.29; −.04]	—
Group x subj. age (prop. score)	—	—	−.07 [−.22; .07]	—	—	.05 [−.07; .18]
*R* ^2^	.13	.13	.14	.37	.39	.37

^*^p < .05, ^**^p < .01, ^***^p < .001.

Considering quality of life, a younger subjective age was also associated to quality of life although this time to a smaller degree than cancer status. This association between subjective age and quality of life did not appear to depend on participants’ cancer status. However, following Model 2a, the association between quality of life and the perception of time as a finite resource appeared to depend on the participants’ cancer status. Indeed, a group-stratified analysis could show that in the OA group, a stronger perception of time as a limited resource was associated to an increased quality of life (see Table 4).

When depression as a binary outcome was considered (Table 3), an association to subjective age, however, not to perception of time as a finite resource could be shown. The respective interaction in Model 2b showed that the association between subjective age and depression appeared to depend on cancer status. Based on the group-stratified analysis, a positive association between older subjective age and depression could be shown in the OA group (see Table 4).

**Table 3.** Results from Hierarchical Binary-logistic Regression with Depression as the Outcome.

Predictor	Depression		
	Model 1	Model 2a	Model 2b
	OR [95% CI]	OR [95% CI]	OR [95% CI]
Age	0.97 [0.95; 1.00]	0.99 [0.96; 1.02]	0.99 [0.95; 1.03]
Sex	1.12 [0.59; 2.16]	1.38 [0.68; 2.81]	1.09 [0.56; 2.14]
Family status (alone vs. with partner)	0.91 [0.44; 1.89]	1.05 [0.49; 2.26]	0.89 [0.41; 1.92]
Group (OA vs. OAC)	3.70*** [1.71; 7.98]	0.26 [0.01; 4.08]	2.62* [1.15; 5.97]
Time as a finite resource	1.20 [0.85; 1.70]	0.80 [0.45; 1.41]	1.20 [0.84; 1.71]
Subj. age (prop. score)^a^	1.46* [1.09; 1.95]	1.47* [1.10; 1.97]	3.01** [1.46; 6.21]
Group x Time as finite resource	—	1.99 [0.95; 4.19]	—
Group x subj. age (prop. score)	—	—	0.38* [0.17; 0.83]

^*^p < .05, ^**^p < .01, ^***^p < .001.

^a^Subjective Age was rescaled, so that the odds ratio refers to a 10% change in the subjective age proportional score.

**Table 4.** Group-Stratified Analysis: Older Adults without Terminal Disease Sample (OA) versus Older Adults with Cancer Sample (OAC) and Quality of Life (Linear Regression) as well as Depression (Binary Logistic Regression).

	Quality of life		Depression	
	OA	OAC	OA	OAC
Predictor	ß [95% CI]	ß [95% CI]	OR [95% CI]	OR [95% CI]
Age	−.05 [−.27; .16]	−.13 [−.36; .10]	0.98 [0.94; 1.03]	0.98 [0.95; 1.02]
Sex	−.18 [−.39; .03]	.07 [−.16; .30]	2.46 [0.70; 10.46]	0.86 [0.36; 2.05]
Family status (alone vs. with partner)	.10 [−.11; .30]	.00 [−.22; .22]	0.69 [0.19; 2.61]	1.28 [0.49; 3.35]
Time as a finite resource	.30** [.08; .51]	−.08 [−.31; .14]	0.84 [0.40; 1.83]	1.33 [0.88; 2.06]
Subj. age (prop. score)^a^	−.34** [−.54; −.13]	−.19 [−.42; .03]	3.10* [1.56; 7.46]	1.12 [0.82; 1.56]

*Note.* ß are standardized coefficients from linear regression. OR are odds-ratios from binary logistic regression.

^*^p < .05, ^*^^*^p < .01.

^a^Within the binary logistic regression (outcome: depression) subjective age was rescaled, so that the odds ratio refers to a 10% change in the subjective age proportional score.

## Discussion

Research that examines whether the experience of cancer in the latter years of one’s life has any association with perception of time and with subjective evaluation of age is scarce. We hypothesized that older adults with life-threatening cancer experience the time remaining to them more as a limited resource than will controls and that the subjective age of older cancer patients and the percentage of those who feel the same age or older than their chronological age will be higher than for controls. We also expected that higher scores in appreciation of one’s lifetime as being a finite resource and having a higher subjective age will be associated with heightened distress, lowered quality of life, and higher levels of depression in older adults both with and without cancer. The findings on these hypotheses take on particular importance in these times of rapidly aging societies and consequently of increasing significance for geriatric oncology.

On the topic of perception of time, our results reveal, as hypothesized by the socioemotional selectivity theory, that older cancer patients perceive their remaining lifetime more intensively as a limited resource than do older adults not suffering life-threatening disease (see also Fitzpatrick et al., 1980). Given the severity of their disease and its terminal prognosis, it seems indeed unsurprising that one’s remaining time is more intensively experienced as a limited resource in our sample of older participants with advanced cancer as compared to older adults not suffering from severe disease. Cancer brings with it a significant additional shortening of perceived future time at a stage of life—that is, later life/old age—that is already marked, as socioemotional selectivity theory predicts, by foreshortened future prospects. Socioemotional selectivity theory also argues that intimate social relations increase radically in importance when the future time available to one begins to be perceived as limited (Carstensen, 2006). Our findings therefore give further support to the idea that the need for and role of intimate social relations is very likely to have more importance for older adults suffering severe cancer (Pinquart & Silbereisen, 2006). Interestingly, however, we could not show an association of cancer with the other dimensions of time perception considered. While previous qualitative research involving a younger sample suggested that patients with terminal disease may have a more past-oriented perception of time (Rovers et al., 2019), this could quantitatively not be shown in our sample. As Strough et al. (2016) noted, a focus on future opportunities considerably decreases with older age. In our study, potential effects of cancer may have been constrained by an age-related shift from long-term to more short-term or current events, which would also explain the overall neutral stance of the sample to the respective item, essentially meaning they neither strongly focused on past nor the future. Previous research has also shown that older adults tend to engage less in rumination (Nolen-Hoeksema & Aldao, 2011; Sütterlin et al., 2012), which may explain that participants irrespective of their cancer status gave likewise rather neutral answers to preoccuation with time as a current concern or their use of time. It is surprising that we could not show that participants with cancer experienced time passing more strongly than those without as in research by van Laarhoven et al. (2011), though it should be noted that the stronger generalization in the framing of our item may have caused this, that is, we asked participants their agreement to the statement that “Time passes faster today than ever before in my life,” while van Laarhoven et al., 2011 asked their participants patients how long the past week had seemed to them.

The hypothesized differences in subjective age experience between the two samples were supported at two levels, that is, in relation to percentages of participants feeling the same age or older than their age on paper and in terms of scores for proportional differences in subjective age. While feeling older than one’s chronological age is a rare occurrence in heterogeneous samples of older adults, with rates typically coming in at lower than 10% (e.g., Rubin & Berntsen, 2006), as much as one third of the OAC group reported to feel older than their age.

Further, previous empirical research suggests that perception of time may be an important predictive factor in psychosocial well-being in that a perspective that anticipates only a foreshortened future is related to lower psychological well-being and physical health (Brothers et al., 2016). Our findings however point to a positive association between the stronger perception of time as a limited resource and increased quality of life in the group of older adults without terminal disease, while no such association could be shown in the OAC group. While this goes against our initial hypothesis, it may in light of previous research still be a valid and conclusive finding: As Gabrian et al. (2017) argued, not just the perception of lifetime as a limited resource has to be considered but also the valence attributed to this perception. In participants without terminal disease, this may not necessarily be a negative one, but a sign of higher value attributed to the time remaining. As one’s lifetime is by nature constrained, more strongly perceiving it in such a realistic way may also support protective mechanisms in the positively selected group of healthy older adults (Lachman et al., 2008).

Empirical research also suggests that subjective age has implications for a range of outcome parameters, including well-being, cognitive functioning, and for health-related occurrences such as longevity (Stephan et al., 2011, 2012). Feeling younger is associated with better health and functioning, as well as improved well-being, whereas feeling older generally accompanies poor health as well as a lower level of functioning and well-being (Baum & Boxley, 1983; Boehmer, 2006; Linn & Hunter, 1979). One study on 159 post-surgical cancer patients (aged between 24 and 86 years) showed that patients who felt either younger or the same age as their chronological age reported better quality of life than those who felt older than their years (Boehmer, 2006). To the best of our knowledge, our study is the first to show that feeling younger continues to be meaningfully related with both better quality of life and less perceived distress in cancer patients and people free of cancer in middle and old age after controlling for confounding factors. For depression, however, an association between younger subjective age and less depression could only be shown in the sample of participants without cancer. Given the limited sample sizes, it may however well be that our study was underpowered to detect a possibly attenuated effect in participants with cancer. Future research with larger samples might show that a younger subjective age is indeed related to depression also in older cancer patients.

Interpreting this set of findings, the first important consideration is to note as a research and clinical issue that chronological age—we considered a broad age range from 50 to 92 years—appeared to play a less pronounced role in predicting distress, depression, and quality of life in regression models. We interpret this finding as an initial indication that chronological age might not provide a very reliable guideline in clinical contexts to explain differences in clinical outcomes among middle-aged and older cancer patients. The result also, at least indirectly, supports the idea that the negative age stereotyping that automatically links higher chronological age to less positive psychosocial outcomes is not necessarily well-founded in relation to aging in general and not in the specific case of older cancer patients either (Chang et al., 2020).

A somewhat contrasting conclusion that may be drawn, however, is that our data suggest that it might be worthwhile dedicating some more clinical attention to the issue of subjective age when it comes to, that is, quality of life. Certainly, our cross-sectional data do not allow us to assume any causal role, but past *longitudinal* research has indeed provided some support for the presence of such causality, in the sense that older subjective age does indeed influence lower health and well-being, while the case for causal arrow being pointed in the opposite direction is less clearly supported in previous research (Spuling et al., 2013). Longitudinal research in OACs could test bidirectional effects between subjective age and clinical outcomes to better understand the implications of our cross-sectional findings. Yet, one cautious practical recommendation would be to suggest that it could be helpful to the psychosocial adaptation of older patients suffering advanced cancer to address in psychosocial consultations with such patients how they perceive and evaluate their aging process in general. As Supplementary Figure 2 suggests, having to confront cancer may in some subgroups of older cancer patients trigger a particularly strong developmental identity transition toward “feeling older,” which may further undermine their perceived quality of life in their day-to-day lives. Clinical consultation may be able to direct the focus of self-awareness toward what remains possible in the lives of patients who now “feel very old.”

Again, we can only speculate as to why experiencing time as a limited resource played a less important role in the multivariate model. One explanation might be that we only used a 1-item measure that we specifically constructed to be short, in recognition of the vulnerability of our group of advanced cancer patients. In contrast to 1-item measures such as subjective age, where validity is supported by a vast previous literature (e.g., Rubin & Berntsen, 2006), the instrument that we used to record perception of time was newly developed for this study and should therefore be treated with caution. On the other hand, given that our 1-item indicators are driven by established theoretical approaches used in general in time-related research (Friedman & Janssen, 2010; John & Lang, 2015; Wittmann & Lehnhoff, 2005) and in particular in time-related cancer research (van Laarhoven et al., 2011; Zhou et al., 2018), one might see the procedure as unproblematic in research where, as in this study, older adults with life-threatening cancer are included.

Several limitations of our study should be considered. As mentioned above, our cross-sectional design does not allow one to come to any causal conclusions. In addition, sample sizes were relatively small, though it should be noted that recruiting a sample of older adults with advanced cancer near the end of life represents quite a challenge and that 3-digit samples are not easy to achieve. That we applied only 1-item measures for assessing time-related factors (with only a five level Likert scaling) and that we employed very short scales for clinical outcomes also limit the reliability and validity of our data, which might have negatively influenced especially the detection of smaller effects between the groups. However, we did make an attempt to provide a straightforward conceptual justification for our choice of the five items we selected. Unfortunately, our data protocol was severely constrained in its options for choosing more established measures, such as Carstensen and Lang’s (1996) Future Time Perspective Scale or Zimbardo and Boyd’s (2015) Time Perspective Inventory, due to the extreme vulnerability of the OAC group, which required us to reduce the data-collection burden on them to a minimum. Furthermore, we were unable to ensure a rigorous matching of the two samples in our study: although we did control for a number of potential confounding factors, such procedures have their limits due to the size of our samples. Finally, it has to remain open whether the associations between subjective age and our outcomes are cancer specific or would have been observed also in other major diseases (Schönstein et al., 2021).

In conclusion, our study provides support for the idea that there is a general need for investment in future research and for closer liaison between the disciplines of geriatric oncology and geropsychology. Important issues for future research in the area might, for example, be to examine whether subjective age has any relationship with the survival times of older cancer patients. The existence of this link has found considerable support in previous research on the role of subjective age in survival in older populations in general (Levy et al., 2002).

## Supplemental Material

sj-pdf-1-jah-10.1177_08982643211063162 – Supplemental Material for Experience of Time and Subjective Age When Facing a Limited Lifetime: The Case of Older Adults with Advanced Cancer Click here for additional data file. Supplemental Material, sj-pdf-1-jah-10.1177_08982643211063162 for Experience of Time and Subjective Age When Facing a Limited Lifetime: The Case of Older Adults with Advanced Cancer by Katsiaryna Laryionava, Anton Schönstein, Pia Heußner, Wolfgang Hiddemann, Eva C. Winkler and Hans-Werner Wahl in Journal of Aging and Health

## Note

1. We decided against the existing Future Time Perspective Scale (Carstensen & Lang, 1996), because some items seemed ethically problematic for use with the OAC group (e.g., “My future is filled with possibilities”). Aside from that judgment, we considered other scales, such as Zimbardo and Boyd’s Time Perspective Inventory (2015), to be too long to be appropriate with the OAC group.
