# Supplementary material for: Experience of Time and Subjective Age When Facing a Limited Lifetime: The Case of Older Adults with Advanced Cancer
Source: J Aging Health. 2021 Dec 30;34(4-5):736–49. doi: 10.1177/08982643211063162 (PMC9446453; doi:10.1177/08982643211063162)
Supplement: sj-pdf-1-jah-10.1177_08982643211063162 – Supplemental Material for Experience of Time and Subjective Age When Facing a Limited Lifetime: The Case of Older Adults with Advanced Cancer [file sj-pdf-1-jah-10.1177_08982643211063162.pdf]

Supplementary Table 1

Descriptive Comparison of the two Study Groups (Older Adults with Advanced Cancer vs. Older Adults without Life-Threatening Disease).

	Overall (N= 181)	OAC (n=92)	OA (n=89)	*p*-Value[a]
Age	69.48 (10.17)	70.95 (8.5)	67.98 (11.5)	.051
Sex				.270
Male	96 (53%)	53 (58%)	43 (48%)	
Female	85 (47%)	39 (42%)	46 (52%)	
Marital Status				> 0.999
Separated/ Widowed / Alone	47 (27%)	24 (26%)	23 (27%)	
Married and living together	130 (73%)	68 (74%)	62 (73%)	

Note: [a] Welch two sample t-test for age as a continuous variable, and Pearson's Chi-squared test for sex and marital status as categorical variables.

Supplementary Table 2

Number and Percentage of Missing Values Across the Major Study Variables.

Variable	Missings n (%)
Age	1 (< 1 %)
Sex	0 (0%)
Marital Status	4 (2%)
Subj. Age (prop. Score)	5 (3%)
“Lifetime lim. res.”	0 (0%)
“Future vs. past”	0 (0%)
“Passing of time”	1 (< 1 %)
“Time as current conc.”	0 (0%)
“Using time”	0 (0%)
Subj. Age (prop. score)[a]	5 (3%)
Distress	2 (1%)
Self-assessed Quality of Life	1 (< 1 %)

Note: [a]Beyond these missings three additional observations (outliers, further than 3 SDs from the mean) were set to missing.

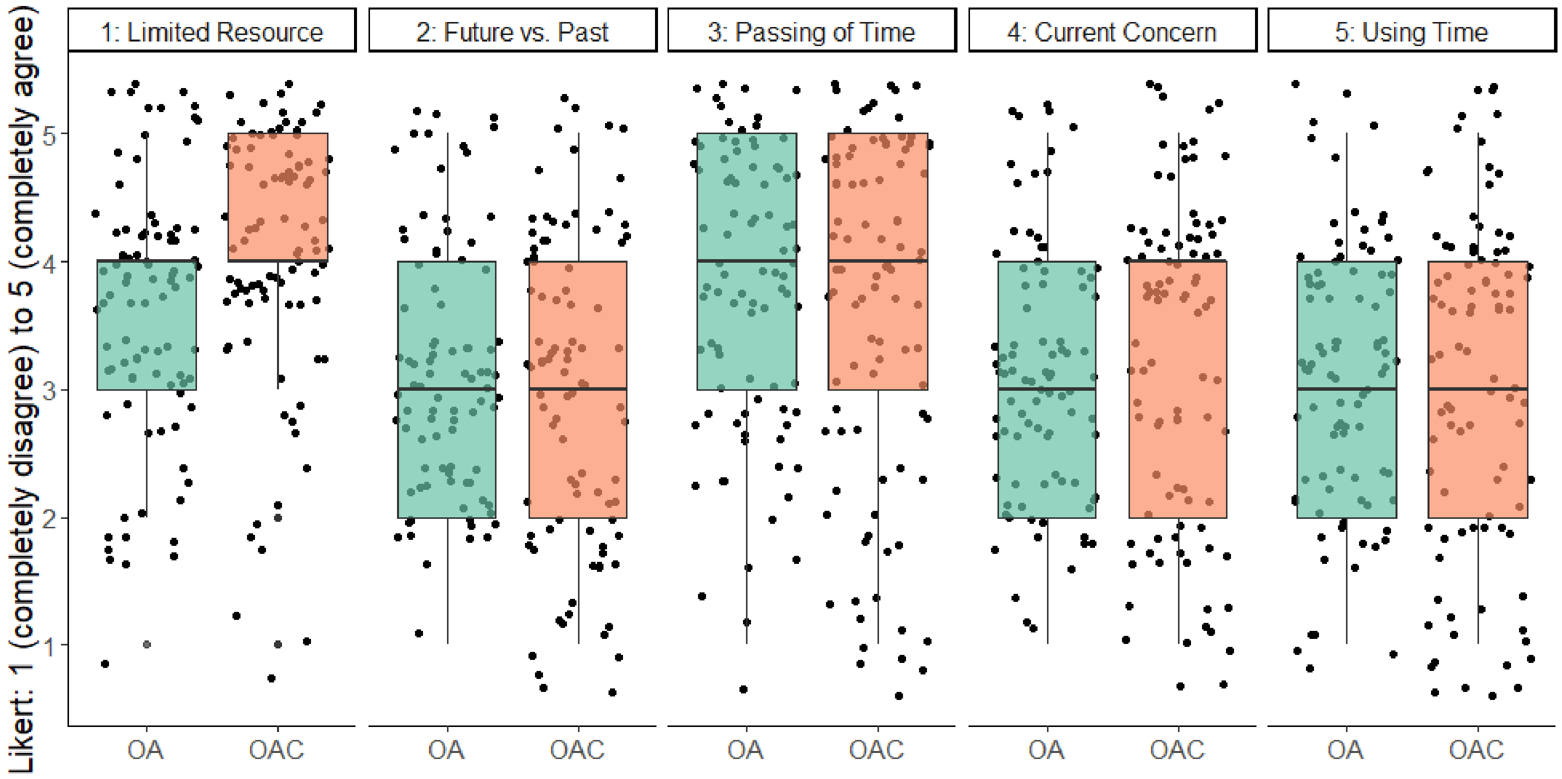

Supplementary Figure 1. Descriptive comparison between the older adults without terminal disease group (OA, green) and the older adults with cancer group (OAC, red) across agreement (y-axis) to time related variables. Dots represent individual observations which are also summarised by boxplots. Note that to avoid overplotting individual observation are jittered using random noise and can originaly by definition of the Likert-scale only take integer values.

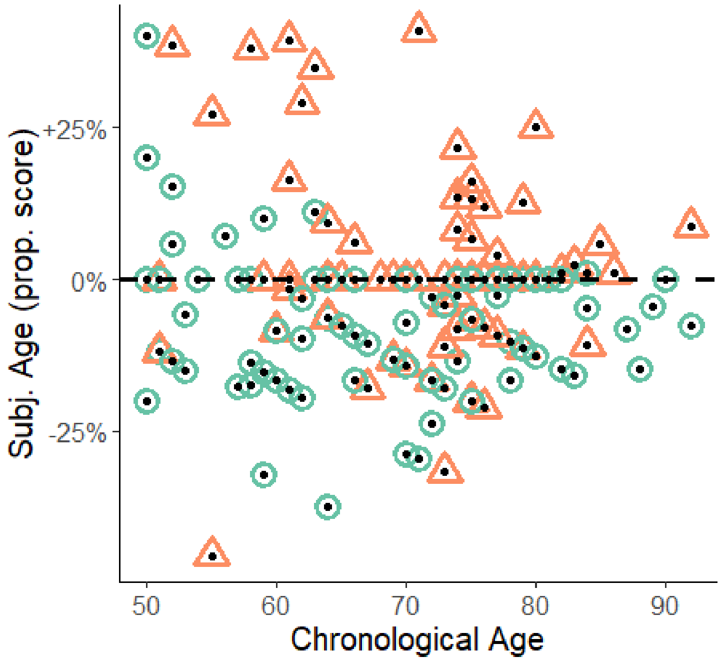

Supplementary Figure 2. Scatterplot displaying participants' subjective age (y-axis) as proportional score depending on their chronological age (x-axis) in years. Circles (green) are older adults without life threatening disease (OA) and triangles (red) are older adults with cancer (OAC). Univariate subjective age outliers excluded; available cases set.
